# The Peroxidase/H_2_O_2_ System as a Free Radical-Generating Agent for Gelling Maize Bran Arabinoxylans: Rheological and Structural Properties

**DOI:** 10.3390/molecules16108410

**Published:** 2011-10-10

**Authors:** Ana L. Martínez-López, Elizabeth Carvajal-Millan, Jaime Lizardi-Mendoza, Yolanda L. López-Franco, Agustín Rascón-Chu, Erika Salas-Muñoz, Cécile Barron, Valérie Micard

**Affiliations:** 1 CTAOA, Laboratory of Biopolymers, Research Center for Food and Development, CIAD, AC., Hermosillo, Sonora 83000, Mexico; 2 CTAOV, Research Center for Food and Development, CIAD, AC., Hermosillo, Sonora 83000, Mexico; 3 Chemistry Faculty, Autonomous University of Chihuahua, Chihuahua 31125, Mexico; 4 U.M.R. Ingénierie des Agropolymères et des Technologies Emergentes. SupAgro / INRA, 2 Pl Viala Montpellier 34060 cedex 01, France

**Keywords:** arabinoxylan gels, free radicals, oxidative cross-linking

## Abstract

The oxidative gelation of maize bran arabinoxylans (MBAX) using a peroxidase/H_2_O_2_ system as a free radical-generating agent was investigated. The peroxidase/H_2_O_2_ system led to the formation of dimers and trimer of ferulic acid as covalent cross-link structures in the MBAX network. MBAX gels at 4% (w/v) presented a storage modulus of 180 Pa. The structural parameters of MBAX gels were calculated from swelling experiments. MBAX gels presented a molecular weight between two cross-links (Mc), a cross-linking density (*ρ*_c_) and a mesh size (*ζ*) of 49 × 10^3^ g/mol, 30 × 10^−6^ mol/cm^3^ and 193 nm, respectively.

## 1. Introduction

Gels are three-dimensional polymer networks capable of imbibing large amounts of water [1]. The swelling capacity of gels is directly related to their chemical structure, molecular conformation and cross-linking degree of the polymer network. Covalently cross-linked gels generally present high water absorption capacity, absence of pH or electrolyte susceptibility and exhibit no syneresis after long periods of storage [2]. An example of covalently cross-linked gels is the product of oxidative coupling of ferulated arabinoxylans chains [3]. Arabinoxylans (AX) are important cereal non-starch polysaccharides constituted of a linear backbone of β-(1→4)-linked D-xylopyranosyl units to which α-L arabinofuranosyl substituents are attached through O-2 and/or O-3 [2]. Some of the arabinose residues are ester-linked on (O)-5 to ferulic acid (FA, 3-methoxy-4 hydroxycinnamic acid) [4]. One of the most important properties of AX is the ability to form gels by covalent cross-linking involving FA oxidation by either chemical (ferric chloride, ammonium persulphate) or enzymatic (peroxidase/H_2_O_2_, laccase/O_2_) free radical-generating agents [2,5,6,7]. This oxidation allows the coupling of AX chains through the formation of dimers and trimers of FA (di-FA, tri-FA), generating an aqueous three-dimensional network. The content of covalent bonds in the gel is determined by the extent of oxidative coupling of FA and can be quantified by the formation of di-FA and tri-FA [7]. Five isomeric forms of di-FA structures have been reported in AX gels: 5-5'-, 8-5'-benzo-, 8-*O*-4'-, 8-5'- and 8-8'- [8] and only one tri-FA: 4-*O*-8', 5-5'- [9]. In addition to covalent bonds (di-FA and tri-FA), physical interactions between AX chains can contribute to the gelation process [6,7].

The rheological and structural properties of AX gels induced by laccase have been previously investigated in AX extracted from wheat endosperm and maize bran [10,11]. Recently, the high deformation rheological properties of maize bran AX gels prepared by peroxidase/H_2_O_2_ oxidative cross-linking have been reported [12]. However the small deformation rheological properties and the structural properties of maize bran AX gels generated by peroxidase/H_2_O_2_ system as cross-linking agent have not been reported elsewhere. In the present study, peroxidase/H_2_O_2_ system induced maize bran arabinoxylans gels were formed and their viscoelastic (storage and loss moduli) and structural properties were investigated.

## 2. Results and Discussion

### 2.1. Gelation of MBAX

**Figure 1:**
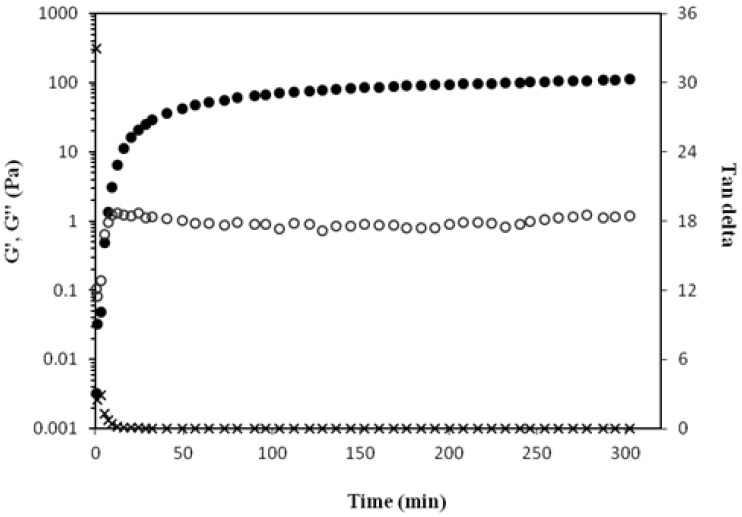
Monitoring the tan δ (X), storage (G'●) and loss (G''○) modulus of MBAX solution during gelation by peroxidase/H_2_O_2_ system at 25 °C, 0.25 Hz and 5% strain. Gels at 4% (w/v) in MBAX.

The formation of MBAX gel over time was rheologically investigated by small amplitude oscillatory shear. Figure 1 shows the development of storage (G') and loss (G'') modulus of a 4% (w/v) arabinoxylan solution undergoing oxidative gelation by the peroxidase/H_2_O_2_ system. The gelation profile followed the characteristic kinetics, with an initial increase of G' followed by a plateau region. The values of G' and G'' at the plateau region were 180 and 2 Pa, respectively. Similar profiles have been previously obtained with wheat water-extractable and water-unextractable arabinoxylans treated with peroxidase/H_2_O_2_ system and laccase, respectively [13,14]. Another study [14] reported a similar G' value at plateau (300 Pa after gelation of 5% wheat AX solutions). The tan δ (G''/G') values decreased during MBAX gelation (Figure 1) indicating the formation of a more elastic material [15].

**Figure 2:**
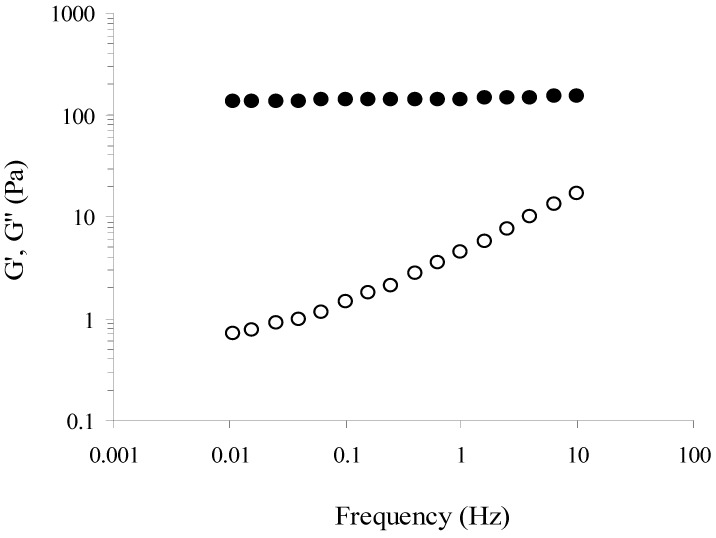
Mechanical spectrum of MBAX gels at 6 h (G'●, G''○).Gels at 4% (w/v) in MBAX. Data obtained at 25 °C and 5% strain.

The mechanical spectrum of MBAX gels after 6 h gelation was typical of solid-like materials with a linear G' independent of frequency and G'' much smaller than G' and dependent on frequency (Figure 2). This behavior is similar to that reported for arabinoxylans from maize [11] and wheat induced by laccase [6].

Ferulate monomer and total di-FA and tri-FA contents of the sample were measured before and after 6 h of gelation (Table 1). Ferulic acid was oxidized (81% of initial FA content) during the gelation process. After gelation the di-FA content in MBAX did not increase, but rather decreased from 0.14 to 0.03 µg/mg MBAX, respectively. The tri-FA was present only in trace quantities (0.003 µg/mg MBAX). Nevertheless, the tan δ (G''/G') values confirm the formation of a true gel after peroxidase/H_2_O_2_ treatment. These results could be related to the formation of ferulated cross-linking structures which cannot be released by mild alkaline hydrolysis and/or to physical interactions between arabinoxylan chains. A decrease in FA content without a proportional formation of di-FA and tri-FA structures has been reported before in laccase and peroxidase/H_2_O_2_ systems induced arabinoxylans gels [16,17]. The predominant dimers in MBAX gels were 8-5' (72%) and 5-5' (20%). The 8-*O*-4' di-FA structure accounted for only 4% of total dimer content. A previous research on maize bran arabinoxylan gels induced by peroxidase/H_2_O_2_ system reported the 8-8' di-FA structure as predominant [17]. In the present study, the 8-8' di-FA structure was not detected.

**Table 1:** Characteristics of MBAX before and after 6 h gelation.

	t = 0 h	t = 6 h
FA (μg/mg MBAX)	0.255 ± 0.017	0.049 ± 0.002
di-FA (μg/mg MBAX)	0.135 ± 0.011	0.03 ± 0.001
tri-FA (μg/mg MBAX)	0.064 ± 0.010	Traces
Mc^a^ ×10 ^3^ (g/mol)	-	49 ± 3.0
*ρ*_c_^b^ × 10^−6^ (mol/cm^3^)	-	30 ± 2.0
*ζ*^ c^ (nm)	-	193 ± 10.0

^a^ Molecular weight between two cross-links; ^b^ Cross-linking density; ^c^ Mesh size; All values are means ± standard deviation of three repetitions.

### 2.2. Swelling and Structure

The equilibrium swelling of MBAX gels was reached between 8–15 h. The swelling ratio value (q) in MBAX gels at 4% (w/v) was 43 g water/g MBAX, which is higher than the q value reported elsewhere [11] for 3.5% (w/v) MBAX gels induced by laccase (20 g water/g AX). The higher water uptake of gels made at 4% in MBAX could be explained in terms of the existence of longer uncross-linked AX chains sections in the network as the covalent cross-links (di-FA, tri-FA) contents in this gel is similar to that reported in MBAX gel al 3.5%. Uncrosslinked polymer chains sections in the gel can expand easily, leading to higher water uptake [18].

The molecular weight between two cross-links (Mc), the cross-linking density (ρc) and the mesh size (*ζ*) values of the different MBAX gels are presented in Table 1. These values were different to those reported before in maize bran arabinoxylans gels induced by laccase (20 × 10^3^ g/mol, 75 × 10^−6^ mol/cm^3^ and 48 nm for Mc, ρc and *ζ*, respectively) [11]. As the covalent cross-links (di-FA, tri-FA) content in these studies were similar, some differences in the involvement of physical interactions between AX chains and/or possible higher oligomers of ferulate in the final MBAX gel structure could be responsible of these results. Higher mesh sizes values (201–331 nm) have been reported in laccase induced wheat flour AX gels at lower polysaccharide concentrations (0.5–2% w/v) [10] and higher di-FA and tri-FA contents. The latter could be related to the high molecular weight reported in AX from wheat flour (438 kDa) [10] in comparison to the alkali-extracted AX from maize bran (197 kDa) used in the present study.

Clearly, complementary studies using the same arabinoxylans extract but different cross-linking enzyme(s) are needed to establish a relationship between the enzymatic free radical-generating agent and the arabinoxylan gel viscoelastic and structural properties.

## 3. Experimental

### 3.1. Materials

Maize bran arabinoxylans (MBAX) were obtained and characterized as previously reported [19]. MBAX contain 85% dry basis (d.b.) of pure AX. MBAX presented a ferulic acid (FA), di-FA, and tri-FA content of 0.25, 0.14, and 0.07 μg/mg of MBAX, respectively, and an A/X ratio of 0.72. Horseradish peroxidase (POD, donor: Hydrogen-peroxide oxidoreductase, EC 1.11.1.7) type I and all other chemical products were purchased from Sigma Chemical Co. (St Louis, MO, USA).

### 3.2. Methods

#### 3.2.1. Preparation of MBAX Gels

The reaction mixtures contained MBAX solution at 4% (w/v) in 0.05 M citrate phosphate buffer pH 5, peroxidase (1.670 PU/mg of MBAX) and H_2_O_2_ (1.5 µmol/mg of MBAX). Gels were allowed to form for 6 h at 25 °C.

#### 3.2.2. Rheological Tests

The formation of the MBAX gel was followed using a strain-controlled rheometer (ARES 2000, Rheometric Expansion System, Rheometric Scientific, Champ sur Marne, France) in oscillatory mode as follows: cold (4 °C) solutions of 4% (w/v) MBAX were mixed with peroxidase/H_2_O_2_ and immediately placed in the cone and plate geometry (5.0 cm in diameter, 0.04 rad in cone angle) maintained at 4 °C. MBAX gelation kinetic was started by a sudden increase in temperature from 4 to 25 °C and monitored at 25 °C for 6 h by following the storage (G') and loss (G'') modulus and tan δ (G''/G'). All measurements were carried out at a frequency of 0.25 Hz and 5% strain (linearity range of visco-elastic behavior). Frequency sweep (0.01–10 Hz) was carried out at the end of the network formation at 5% strain and 25 °C.

#### 3.2.3. Phenolic Acids Content

FA, di-FA and tri-FA contents in MBAX gels were quantified by reverse phase high-performance liquid chromatography (RP-HPLC) after a deesterification step, as described elsewhere [6]. An Alltima (Alltech, Deerfield, IL, USA) C18 column (250 × 4.6 mm) and a photodiode array detector Waters 996 (Millipore Co., Milford, MA, USA) were used to record the ferulic acid and its di-FA and tri-FA spectra. Detection was by UV absorbance at 320 nm. Gradient elution was performed using acetonitrile and sodium acetate buffer 0.05 M, pH 4.0, at 1 mL/min at 35 °C, in linear gradients from 15:85 to 35:65 in 30 min, from 35:65 to 60:40 in 0.5 min, from 60:40 to 15:85 in 4.5 min, and finally maintained at 15:85 for 5 min.

#### 3.2.4. Swelling

After peroxidase/H_2_O_2_ system addition, MBAX solutions were quickly transferred to a 2 mL tip-cut-off syringe (diameter 1.5 cm) and allowed to gel for 6 h at 25 °C. After gelation, the gels were removed from the syringes, placed in glass vials and weighted. The gels were allowed to swell in 20 mL of 0.02% (w/v) sodium azide solution to prevent microbial contamination. During 36 h the samples were blotted and weighed. After weighing, a new aliquot of sodium azide solution was added to the gels. Gels were maintained at 25 °C during the test. The equilibrium swelling was reached when the weight of the samples changed by no more than 3% (0.06 g). The swelling ratio (q) was calculated by Equation (1):

q = (W_s_ − W_MBAX_)/W_MBAX_ (1)

where W_s_ is the weight of swollen gels and W_MBAX_ is the weight of MBAX in the gel [7].

#### 3.2.5. Structure

From swelling measurements, the molecular weight between two cross-links (Mc), the cross-linking density (ρc) and the mesh size (ζ) values of the different MBAX gels were obtained as reported elsewhere [7]. Mc was calculated using the model of Flory and Rehner (1943) [20] modified by Peppas and Merrill [21] for gels where the cross-links are introduced in solution [Equation (2)]:

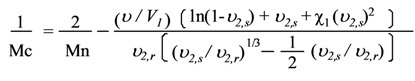
(2)
where Mn is the number average molecular weight of MBAX (considering only the xylose backbone). The Mn value was calculated from the MBAX molecular weight value knowing the A/X ratio. In Equation (2), V_1_ is the molar volume of water (18 cm^3^/g), u_2,r_ and u_2,s_ are the polymer volume fraction of the gel in a relaxed state (directly after gel formation) and at equilibrium swelling, respectively. u_2,r_ and u_2,s_ were calculated from the weight of the gels before exposure to the sodium azide solution and at equilibrium swelling, respectively. χ_1_ is the Flory polymer-solvent interaction parameter. The arabinoxylans-water system was calculated to correspond to theta conditions [22]. χ_1_ was therefore taken as equal to 0.5 for the present system.

After Mc calculation by Equation (2), the average mesh size (ζ) of the MBAX gels was obtained as reported elsewhere [1] [Equation (3)]:

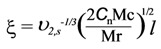
(3)
with Mr representing the molecular weight of the repeating unit (xylose, 132 g/mol), Cn the characteristic ratio for arabinoxylans (11.5) [23] and *l* the bond length between two xyloses (0.286 nm). The cross-linking density in MBAX gels (ρc) has been calculated from Mc as reported elsewhere [24] [Equation (4)]:

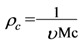
(4)


#### 3.2.6. Statistical Analysis

All measurements were made in triplicate and the coefficients of variation were lower than 8%. Results are expressed as mean values.

## 4. Conclusions

MBAX gels can be induced by peroxidase/H_2_O_2_ system as free radical-generating agent. At the end of gelation ferulic acid is oxidized but no increase in known covalent cross-linking (di-FA, tri-FA) content was obtained. MBAX gels presented a high elasticity suggesting the implication of ferulate covalent cross-links structures others than di-FA and tri-FA and/or physical interactions to the MBAX gel structure. Several questions remained to be elucidated, especially those concerning the formation of different ferulate cross-linking structures and their contribution to the gel development and properties. Further research is undergoing in order to explore this relationship.
